# Pluripotent stem cell derived hepatocytes: using materials to define cellular differentiation and tissue engineering

**DOI:** 10.1039/c6tb00331a

**Published:** 2016-05-06

**Authors:** B. Lucendo-Villarin, H. Rashidi, K. Cameron, D. C. Hay

**Affiliations:** a Medical Research Council Centre for Regenerative Medicine , University of Edinburgh , 5 Little France Drive , Edinburgh , EH16 4UU , Scotland , UK . Email: davehay@talktalk.net ; Tel: +44(0)1316519500

## Abstract

Pluripotent stem cell derived liver cells (hepatocytes) represent a promising alternative to primary tissue for biological and clinical applications. To date, most hepatocyte maintenance and differentiation systems have relied upon the use of animal derived components. This serves as a significant barrier to large scale production and application of stem cell derived hepatocytes. Recently, the use of defined biologics has overcome those limitations in two-dimensional monolayer culture. In order to improve the cell phenotype further, three-dimensional culture systems have been employed to better mimic the *in vivo* situation, drawing upon materials chemistry, engineering and biology. In this review we discuss efforts in the field, to differentiate pluripotent stem cells towards hepatocytes under defined conditions.

## Abstract 2

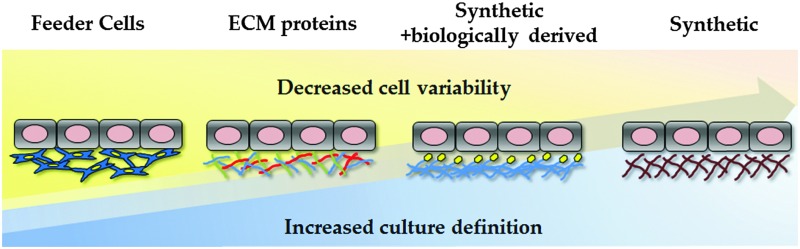
Pluripotent stem cell derived liver cells (hepatocytes) represent a promising alternative to primary tissue for biological and clinical applications.

## 1. Introduction

The advent of human pluripotent stem cells (hPSCs) and their efficient differentiation allows users to custom-make human tissue ‘in a dish’. This has major implication in biomedicine and will likely lead to personalised regenerative medicines of the future. Our particular interest is in the liver, and the generation of functional tissue from human pluripotent stem cells. The major cell type of the liver is the hepatocyte and we, and others,^1^ have been working to produce these cells at the scale for basic research and therapeutic purposes. While freshly isolated human hepatocytes represent the current gold standard,^2,3^ they are a scarce and expensive resource with variable performance. The isolation of primary hepatocytes commences with collagenase digestion of the liver followed by density-gradient centrifugation.^4^ Post-isolation, hepatocyte phenotype is lost and cells begin to senesce, limiting their widespread use.^5–9^ In an effort to preserve the cell phenotype, a number of approaches have been developed, including the modification of culture media, the use of different extracellular matrices, and the development of co-culture formats.^5,7,10,11^ Despite the advantages of these approaches, phenotypic instability still hinders the routine use of primary human hepatocytes.^12^ As a consequence, alternative models have been developed to study human liver biology and model cell based therapy. Those include the use of human cancer cell lines, and animal derived hepatocytes.^1^ While these cell types are promising, they also suffer from limitations which limit their routine deployment. These include genomic instability,^13^ incomplete gene expression,^14–17^ scale-up limitation,^18^ heterogeneous culture and species differences.^19^

While the field faces major challenges, progress is being made. Recent studies provide hope that some of the previous limitations associated with hepatic progenitor cell isolation and expansion have been addressed. Hepatic progenitor cells (HPCs), possess the capacity to regenerate liver epithelia. Although HPCs are extremely rare in healthy liver, their scalability and plasticity makes them an attractive cell source of hepatocytes for application. Recently, Lu *et al.* isolated a defined population of HPCs from the mouse liver. The resulting cells were expandable and displayed stability following long term maintenance *in vitro* and *in vivo.* ^20^

Recently, the limited proliferative capacity of adult human hepatocytes has been studied by Levy *et al.* The authors created an oncostatin M dependent expansion system for primary hepatocytes using human papilloma virus oncoproteins.^21^ We have also studied hepatocyte expansion, differentiation and stabilisation using hPSC-derived hepatocyte-like cells (HLCs). In these experiments HLC stability was maintained for over twenty days, revealing a novel gene signature associated with a stable hepatocyte phenotype. Importantly, these findings were successfully translated to GMP grade hESC lines promising therapeutic application in the future.^22^ Most recently, we have employed recombinant laminins to drive hepatocyte differentiation and self-organisation of HLCs from hESC lines available at GMP grade.^23^

We believe that the development of defined culture systems, and novel tissue engineering processes are essential for the delivery of stable, scalable and functional human liver tissue and this is discussed in the review.

## 2. Pluripotent stem cells

**Fig. 1:**
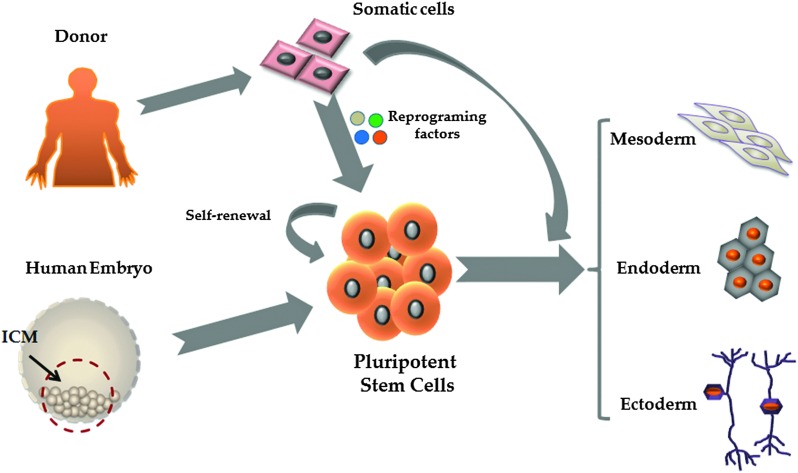
Sources and properties of human pluripotent stem cells (hPSCs). hPSCs can be derived either from somatic cells isolated from the human body or from cells isolated from the inner cell mass (ICM) of human embryos. Pluripotent stem cells possess two important attributes: the ability to self-renew and the capacity to differentiate into any cell type in the human body. Somatic cells can be generated from pluripotent stem cells using multistep differentition procedures or through the use of transcription factor combinations.

Pluripotent stem cells (PSCs) are defined as cells which give rise to all somatic cell types found in the body. Human embryonic stem cells (hESCs) and the more recently described induced pluripotent stem cells (iPSCs) represent the two major sources of pluripotent stem cells (Fig. 1).^24–26^ Human embryonic stem cells are derived from the inner cell mass of blastocyst stage embryos which are not suitable for human implantation.^27^ Pioneering studies of mouse ESCs^28,29^ and of culturing techniques developed in non-human ESC lines^30,31^ and EC (embryonal carcinoma) lines^32^ led to the isolation and propagation of hESC lines for the first time.^27^ While hESCs are highly promising for the field, they have raised ethical issues. In 2006 and 2007 Shinya Yamanaka's laboratory, inspired by the successes in mammalian nuclear transfer,^33^ delivered a PSC population from somatic cells, in a process called reprogramming.^34,35^ In these studies the authors used a core set of transcription factors (Oct 4, Sox2, Klf4 and c-Myc) to reprogram somatic cells to a pluripotent state. Today, PSCs serve as an important resource to generate human tissues.

## 3. Transdifferentiation

In addition to reprogramming, other groups have developed a process called transdifferentiation. This is the direct reprogramming of somatic cells into another type of somatic cell, by-passing the requirement for pluripotency.^36^ Traditional methods of transdifferentiation rely on the expression of a single or a cocktail of tissue specific transcription factors to specify somatic cell types that are representative of all three germ layers.^37–47^

## 4. Hepatocyte differentiation

During liver development various cell types and signalling pathways orchestrate the formation of a highly organised, functional and regenerative organ. The liver acinus represents the basic functional unit of the liver. During acinar formation, the extracellular matrix plays an important role in cell organisation and function and its composition varies depending on the zone of the liver.^48–51^ Therefore, many factors must be considered if one wishes to generate HLCs from human PSCs or from other stem cell populations. To date, hepatocyte differentiation from PSCs has been achieved using two different formats, either three-dimensional cellular aggregation or two-dimensional monolayer culture.

### 4.1 Hepatocyte differentiation in three dimensions

Embryoid bodies (EBs) are three-dimensional (3D) aggregates that permit spontaneous differentiation of hPSCs in suspension, aiming to mimic the 3D tissue niche. Seminal studies reported by Lavon *et al.* demonstrated the ability to isolate and purify HLCs using GFP under control of the albumin promoter.^52^ EB formation, tissue specific reporting and FACS were also employed by Duan *et al.* to differentiate, purify and implant stem cell derived hepatocytes *in vivo*.^53^ Following on from this, Basma *et al.* used EB based systems to derive and enrich HLCs, prior to their characterisation *in vitro* and *in vivo*.^54^ The effect of different ECM components on hepatic differentiation has also been studied by several groups. Schwartz *et al.* observed improved endoderm gene expression in hESC-derived EBs on type I collagen coated plates in the presence of fibroblast growth factor 4 (FGF4) and HGF.^55^ Sirahashi *et al.* highlighted the importance of cell maintenance media, ECM substrates, foetal bovine serum (FBS) and growth factors in hepatocyte differentiation.^56^ More recently, Vosough *et al.* developed a scalable 3D differentiation approach in stirred flasks.^57^ While providing a proof of concept, the presence of undefined components in these experiments have complicated large scale cell production and therefore application. We believe that this highlights the need to truly define and simplify HLC differentiation methodology for use with PSCs.

### 4.2 Directed hepatocyte differentiation in two dimensions

To address the issue of heterogeneous cell differentiation in EBs, research has focused on two dimensional differentiation systems. This requires the stepwise addition of key drivers which move the cells from pluripotency, through definitive endoderm to hepatic progenitors and finally functional hepatocytes. Various procedures exist between labs (Table 1), but most draw upon the seminal observations of D'Amour *et al.* In these studies, hESCs were driven efficiently to definitive endoderm using activin A.^58^ Endoderm differentiation has also been achieved in the presence of multiple factors including Activin A, HGF, bone morphogenic protein 4 (BMP4), FGF2, FGF4, glucocorticoids, insulin and all-*trans*-retinoic acid (ATRA) on different extracellular matrices.^59^ The WNT signalling pathway has also been shown to be important in human endoderm specification.^60–62^ Following endoderm specification, hepatic differentiation has been achieved using a number of approaches. Cai and coworkers developed a differentiation protocol mimicking *in vivo* development using, FGF4 and BMP2 followed by HGF, OSM and dexomethasone.^63^ Agarwal *et al.* employed FGF4 and HGF to promote hepatic specification of hESC-derived endoderm on type I collagen. Following this, hepatocyte differentiation and maturation of the endoderm-derived cells were induced using a combination of BSA, FGF4, HGF, OSM and dexamethasone.^4^ Hay *et al.* induced hepatic progenitor specification from hESC-derived endodermal cells by supplementing the media with 1% DMSO prior to induction of hepatocyte differentiation in media supplemented with HGF and OSM.^65–67^ Brolen *et al.* demonstrated hepatic specification using combinations of BMP2 and -4 with FGF1, -2 and -4, followed by a cocktail of different factors including the epithelial growth factor (EGF), insulin, transferrin, ascorbic acid, FGF4, HGF, dexamethasone, dimethyl sulfoxide (DMSO) and OSM.^68^

**Table 1:** Step-wise differentiation of human embryonic stem cells and induced pluripotent stem cells through definitive endoderm with Activin A (AA) to hepatocytes

Substrate	Definitive endoderm induction	Hepatic specification	Hepatic differentiation	Ref.
MEFs	AA (3d)	FGF4, BMP2 (5d)	HGF (5d) + OSM (5d)	Cai *et al.*, 2007^24^
MEFs/collagen I	AA, FBS, KOSR (5d)	FGF4, HGF, KOSR (3d)	BSA, FGF4, HGF, OSM, Dex (2d) + FGF4, HGF, OSM, Dex (9d)	Argawal *et al.*, 2008^64^
Matrigel	AA, Wnt3a (3d)	1% DMSO, 20% KOSR (5d)	HGF, OSM (9d)	Hay *et al.*, 2008^65^
MEFs	AA, FGF2 (5d)	BMP 2/4, FGF1/2 (11d)	EGF, insulin, hydrocortisone, transferrin (28d)	Brolen *et al.*, 2009^68^
MEFs	AA, bFGF (3d)	HGF, DMSO (8d)	Dex (3d)	Basma *et al.*, 2009^54^
Matrigel	AA (5d)	FGF4, BMP2 (5d)	HGF (5d) + OSM (5d)	Si-Tayeb *et al.*, 2010^71^
Matrigel	AA, Wnt3a, (3d); +AA (2d)	1% DMSO, 20% KOSR (5d)	OSM, HGF (5d)	Sullivan *et al.*, 2010^72^
Matrigel	AA, BMP4,FGF2 (3d)	FGF10 (3d)	FGF4, HGF, EGF (8d)	Toboul *et al.*, 2010^73^
Fibronectin	AA, FGF2, BMP4, Ly294002, CHIR99021 (1d) + AA, FGF2, Ly294003 (1d)++AA, FGF2 (1d)	AA (5d)	HGF, OSM (17d)	Rashid *et al.*, 2010^74^
MEFs	AA, Wnt3a, HFG (3d)	1% DMSO, 20% KOSR (5d)	OSM/HGF (7d)	Chen *et al.*, 2012^75^
Matrigel	AA, Wnt3a (3d)	1% DMSO, 20% KOSR (5d)	HGF, OSM (9d)	Szkolnicka *et al.*, 2014;^76^ Rashidi *et al.*, 2016^77^
Matrigel	AA, Wnt3a, HFG (3d)	1% DMSO, 20% KOSR (5d)	OSM, FGF2, insulin, Dex (7d) + OSM, FGF2, insulin, Dex, LCA, MK4 (4d)	Avior *et al.*, 2015^78^
Laminin	AA, Wnt3a (3d)	1% DMSO, 20% KOSR (5d)	HGF, OSM (9d)	Cameron *et al.*, 2015 *et al.* ^23^

Importantly, the hepatocyte differentiation procedures developed in hESCs have been successfully translated to iPSCs.^69^ Song *et al.* reported that iPSC derived HLCs expressed liver cell markers and related functions, including urea production, albumin secretion and cytochrome P450 activity, which were comparable to hESC-derived HLCs.^70^ Si-Tayeb *et al.* described a four step differentiation protocol in low oxygen to obtain functional HLCs with similarities to hESC-derived HLCs.^71^ The differentiation protocol developed by Hay *et al.* was successfully translated to iPSC technologies, with HLCs displaying metabolic activity and secreting liver proteins.^72^ This was followed by Touboul *et al.* who obtained hepatic progenitor cells from hESC-derived definitive endoderm cells upon treatment with FGF10, retinoic acid and SB431542, a pharmacological inhibitor of TGFβ signaling. Hepatocyte differentiation was induced using FGF4, HGF and EGF.^73^ Following on from this, Rashid *et al.* developed a three step differentiation protocol containing Activin A, BMP4 and FGF2 in the presence of glycogen synthase kinase 3β (GSK3β) and phosphoinositide 3 kinase (PI3K) pathway inhibitors. Notably, iPSC derived HLCs using this method were found to recapitulate key pathological features observed in human monogenic liver disease.^74^

### 4.3 Somatic cell transdifferentiation

As described previously, differentiation can be achieved without the need for pluripotency. Sekiya *et al.* transfected murine embryonic and adult fibroblasts with HNF4α plus FoxA1, -A2 or -A3, successfully obtaining induced hepatocytes (iHeps).^79^ Another seminal study, performed by Huang *et al.*, employed mouse tail fibroblasts as their starting cell population. Using lentivirus over-expression of GATA4, HNF1α and Fox3 and inactivated p19^Arf^ they successfully generated iHeps.^37^ The same group recently reported successful reprogramming of human fibroblasts into iHeps by lentivirus expression of FoxA1, HNF1α and HNF4α. The resulting iHeps displayed hepatic function^80^ and excitingly fueled a bio-artificial liver device that improved animal survival followed by acute liver failure.^81^ Therefore, iHeps and HLCs represent a reliable alternative to primary human hepatocytes, holding great potential for biological and clinical applications. These cells have been employed to accurately predict human drug metabolism and drug responses,^82–84^ as a model to study hepatitis C and B viral life cycle,^85–89^ to study the mechanisms behind the drug-induced liver injury^90,91^ and to modulate drug overdose by using non-coding RNAs.^67^ However, improvements in cell fidelity are required.^92^ One key void in current approaches is the niche for driving cell differentiation and maintaining the cell phenotype. This is an important area of focus which requires interdisciplinary research.^22^

## 5. Biomaterials in stem cell biology

**Fig. 2:**
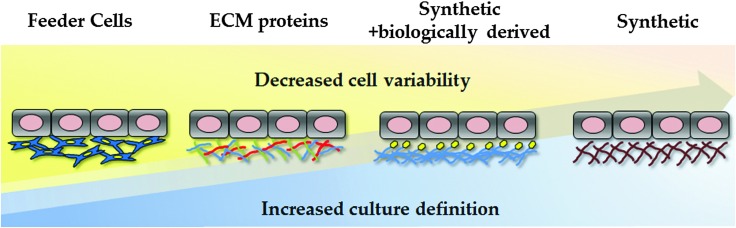
Evolution of human pluripotent stem cell culture and differentiation. Culture conditions are becoming increasingly defined, reducing cell variability and scale-up costs. The field has moved away from animal feeder layers, using undefined ECM formulations, such as Matrigel. As Matrgiel is an undefined mixture of murine proteins and growth factors, the field has now moved toward recombinant proteins and/or synthetic substrates to support hPSC culture and differentiation.

Traditionally, hPSCs were cultured on murine embryonic fibroblast layers or extracellular matrices derived from mice, such as Matrigel. However, the undefined nature of these environments, along with the potential pathogenic and immunogenic issues, is known to be problematic for clinical application and serves as a barrier to reproducible cell based modelling. In an attempt to overcome these limitations, research studies have focused on developing xeno-free and defined substrates that can efficiently replace the use of animal-derived culture components.^23,93–96^ In an attempt to reduce the costs associated with cellular differentiation, attention has also focussed on developing cheap synthetic substrates for PSC maintenance and differentiation (Fig. 2).

### 5.1 Synthetic polymers

Biomaterials can be defined as a material or a combination of materials that can be used to support cells, tissues or organs *in vitro* and *in vivo*. According to their make-up, biomaterials can be classified as polymers, metals, ceramics and composites. Natural and synthetic polymers have gained the attention of researchers in the stem cell field due to their inert nature, diverse composition, capacity to interact with other synthetic or natural substrates, and reliable cost-effective scale-up.

Combinatorial approaches involving the use of synthetic polymers and biologics have been employed in the maintenance and differentiation of hPSCs with promising results. For example, polymers with high acrylate content combined with vitronectin have been reported to successfully maintain the pluripotency properties and colony formation of PSCs for a prolonged period of time.^97^ In addition, to support hPSC maintenance, combinatorial polymer screens have led to the development of hepatic differentiation or co-culture strategies.^98–100^ Examples of successful application of biomaterials in the formation of organoid cultures, include the improved expression of hepatocyte growth factor mRNA with faster formation of viable 3D spheroids following co-culture of hepatocytes with stellate cells on a PLGA substrate.^101^ Increased albumin secretion has also been achieved following the manufacture of endothelialized hepatic spheroids in PDMS honeycomb microwells and poly-l-lactide fibers.^102^ However, batch-to-batch variation, the risk of pathogens from undefined media components and the high cost associated with the production of some biologics compromise technology scale up and application.^105^ Therefore, to deliver somatic cells under defined conditions research has focussed on cheap and scalable synthetic materials and renewable cell sources.

Poly [2-(methacryloyloxy) ethyl dimethyl-(3-sulfopropyl) ammonium hydroxide] (PMEDSAH) is a well-characterised synthetic substrate employed in the maintenance of PSCs. It is thought that the sulfonic group mimics heparin sulphate proteoglycans, which are important extracellular components in hPSC culture systems^99,103–106^ and mesenchymal stem cells derived from hPSCs.^107^ Aminopropylmethacrylamide (APMAAm) represents another example of a synthetic substrate successfully employed in the long-term maintenance of undifferentiated PSCs, by promoting the adsorption of different proteins present in the culture media.^108^ The importance of media protein adsorption in the maintenance of PSCs was also revealed by Mei *et al.*, by identifying a polymer generated from monomers with high acrylate content, displaying the capacity of fixing vitronectin from the culture media and promoting colony formation. This system also revealed the importance of physical parameters of the polymers including wettability, surface topography and surface chemistry.^97^

### 5.2 3D culture systems

**Fig. 3:**
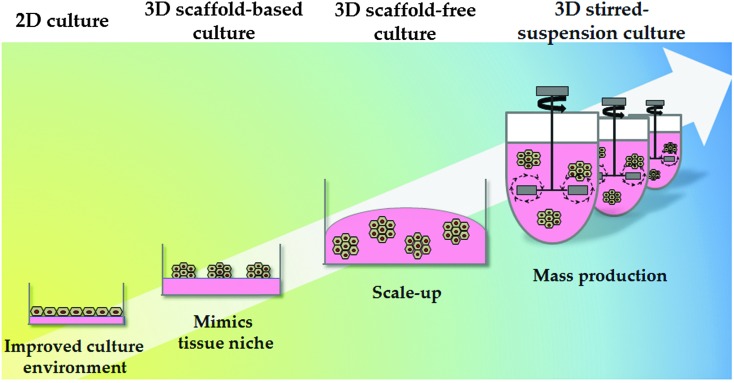
Culture system physical properties. The physical environment of pluripotent and differentiated cells has also evolved; from static 2D culture to 3D culture systems. Static 2D cultures do not accurately mimic the *in vivo* environment and frequently result in alterations in cell biology. 3D culture systems can be divided into scaffold-based and scaffold-free systems. 3D stirred-suspension cultures represent a more advanced culture system, with cells homogeneously exposed to the different factors in the medium. Further research and development is required in this space to permit cost effective technology scale up.

Synthetic polymers have been proven to be economical and effective for the long-term self-renewal, large-scale expansion and direct differentiation of hPSCs. However, these platforms are traditionally applied in two-dimensional (2D) systems. Although 2D systems have proved to be invaluable for studying basic cell biology, cells in these systems are forced to change their cytoskeleton towards flattened shapes, affecting cell-to-cell and cell-to-extracellular environment contacts. These forces lead to reduced polarisation and modification of important signalling pathways, affecting stem cell pluripotency and differentiation.^109^ As a consequence different approaches have been employed to emulate 3D tissue structure. These approaches can be broadly divided into scaffold-based and scaffold-free culture systems (Fig. 3).

#### 5.2.1 Scaffold-based culture systems

Scaffold-based systems rely on the presence of natural or synthetic materials to provide a 3D space for cell attachment. These systems allow the production and organization of cells *in vitro* in a controllable and reproducible manner which is important for routine cell culture. Several materials of synthetic origin have been employed for hPSC maintenance and differentiation in 3D. Hydrogels are an example. Hydrogels are structures composed of cross-linked and hydrophilic polymer units that swell when exposed to water, allowing fine tuning in two or three dimensions. Moreover, hydrogels support the encapsulation and controlled release of bioactive agents and have been used successfully as hPSC culture substrates.^108,110,111^ Of note, Zhang *et al.* identified a thermoresponsive acrylate-based hydrogel for the maintenance enzyme free scale-up of hPSCs under defined conditions.^112^ Although hydrogels can mimic more closely the native 3D environment, the limited diffusion rate of nutrients within the structure can restrict cell type compatibility and long-term cell culture, necessitating cell compatibility testing.^113^

Natural hydrogels have also been employed for cell culture. Alginate, a polysaccharide derived from the cell wall of marine algae, is one example and has been used to successfully culture hepatocytes in 3D. Interestingly, cell aggregation, essential to ensure correct cell function, was mediated by E-cadherin.^114^ In another study, Fang *et al.* demonstrated that exogenous growth factors were required for hepatocyte differentiation *via* EBs in alginate microbeads.^115^ More recently, Jitraruch *et al.* described an intraperitoneal transplantation of alginate-microencapsulated human hepatocytes in a murine model that reduced the severity of liver damage.^116^

#### 5.2.2 Scaffold-free culture systems

In contrast to scaffold-based culture systems, scaffold-free culture systems rely on the self-aggregation of the cells. In hepatocyte biology, tight cell-to-cell contact is important to ensure cell polarisation.^46^ Bioreactors, one of the most studied suspension culture systems, are widely used in chemical and biological industries. In recent years this technology has been translated to the stem cell field, with designs controlling and regulating the cellular microenvironment to support self-renewal and specification of cells. Bioreactors include, rotary cell culture systems and stirred-suspension systems, to create a uniform and dynamic environment for cells.

There are several examples of the scaffold-free culture system applied in hPSC technology. Amit *et al.* developed serum free suspension culture methodology using an IL6R-IL6 (interleukin-6 receptor interleukin-6) fusion protein supporting to support cell expansion and pluripotency.^117^ Steiner employed an alternative approach supporting suspension cultures of hPSCs using neurobasal media supplemented with serum replacement and different ECM components.^118^ More recently, Vosough *et al.* described a stirred-suspension bioreactor culture to obtain iPSC-derived HLCs that post-transplantation supported mice with acute liver damage.^57^

## 6. Current challenges and future directions

Although current differentiation approaches from pluripotent stem cells generate HLCs displaying hepatocyte markers and function, they still express features of foetal hepatocytes. In support of this, Godoy *et al.* compared gene regulatory networks between pluripotent stem cell derived HLCs and adult human hepatocytes. In comparison to PHHs, HLCs expressed the majority of hepatic genes (∼70%). However, there were many genes which did not approach the levels of primary hepatocytes (∼30%). They also identified the expression of colon, fibroblasts and stem cell-associated transcription factors, indicating mixed HLC cultures *in vitro.* ^92^ These studies concluded that *in vitro* culture conditions for primary hepatocytes and HLCs were responsible for their instability and/or not reaching a fully differentiated status. Importantly, those studies provided a ‘blue-print’ to help eliminate non-desired cell traits.

Liver development is a process that extends beyond birth, when there is a switch from placental to enteral nutrition. Fatty acids from breastfeeding become the main energy source, and those are further metabolised by the gut microbiome to secondary metabolites, which lead to liver development in the neonate. In line with this, Avior *et al.* recently demonstrated that a secondary metabolite, lithocholic acid (LCA), or the use of Vitamin K may drive maturation of HLCs. They demonstrated that these additives synergise, regulating the activity of a key nuclear factor, PXR, improving cytochrome P450 enzymes 2C9 and 3A4 expression and function.^127^ While promising, stem cell derived HLC cultures generated using this method are short lived, limiting technology scale up and application.

Searching for defined matrices compatible with maintenance of pluripotency and differentiation capacities of pluripotent stem cells has been traditionally slow and has shown low throughput. However, in recent years, high-throughput (HTP) approaches such as microarraying have allowed rapid screening of chemically diverse substrates that modulate or control cell biology, thus representing an important tool for discovering new materials. HTP technologies have identified polymers with trapping and release properties^119^ for cell isolation, proliferation and differentiation.^120^ Recently, Celiz *et al.* demonstrated that polymer high-throughput screening technology could be used to identify polymer(s) that support stem cell self-renewal and stem cell differentiation to cells representative of the three germ layers. They identified a polymer (HPhMA-*co*-HEMA), which resulted from the polymerization of 5 (*N*-(4-hydroxyphenyl)methacrylamide) and poly (2-hydroxyethyl methacrylate) (polyHEMA), supporting both hPSC culture and pluripotency in a defined environment.^121^ In a separate study a synthetic polyurethane, PU134, was identified employing HTP technology^100^ and facilitated the maturation and maintenance of HLCs from research and GMP grade hESC lines.^22^

Concerning the cell niche, most current differentiation approaches lack many aspects of the micro-environment. Therefore the development of strategies employing relevant cell types from the organ of interest in 3D is required. In this vein, Takebe *et al.* developed an innovative approach. Endothelial and mesenchymal cells, in combination with PSC-HLCs, self-organised into 3D liver-like tissue structures which were functional *in vitro* and *in vivo*. Moreover, the authors demonstrated liver function could be supplied from the mesentery, representing an ideal target site for future cell based therapies.^122^

The emergence of bioprinting in the recent years has also provided new opportunities for the production of printed human liver tissue. Recently Organovo's exVive3D bioprinted liver tissue (Organovo®; USA) has been shown to secrete fibrinogen, albumin and transferrin in proportion to levels observed *in vivo*.^123^ Regarding PSCs, valve-based cell printers have been developed to print viable PSC and HLC populations, and offer the promise of automated tissue manufacture for clinical and research applications.^124^

A common problem associated with most differentiation systems is their static nature. These approaches lack fluid circulation resulting in poor cell perfusion. Recently, Giobbe *et al.* reported a microfluidic system on a chip to differentiate PSCs into HLCs which could predict drug toxicity.^125^ Additionally, Berger *et al.* described a successful microfluidic system employing co-cultures of PSC-derived HLCs and stroma cells, reporting enhanced maturity and polarity.^126^ More recently, we have shown that pluripotent stem cell-derived HLCs can be further improved by exposing them to fluid shear stress.^77^

In conclusion, the ability to derive somatic cell types from hPSCs represents an attractive strategy for human tissue engineering. Despite promising advances in the field, further sophistication is necessary to improve cell type fidelity and stability. To date, differentiation protocols are mainly performed using 2D culture systems lacking the relevant cell types. Recent progress in the field, including; combinatorial approaches and three-dimensional and co-culture strategies provide the promise of more sophisticated systems in the future.
